# The quick Sepsis-related Organ Failure Assessment (qSOFA) is a good predictor of in-hospital mortality in very elderly patients with bloodstream infections: A retrospective observational study

**DOI:** 10.1038/s41598-019-51439-8

**Published:** 2019-10-21

**Authors:** José M. Ramos-Rincón, Adela Fernández-Gil, Esperanza Merino, Vicente Boix, Adelina Gimeno, Juan C. Rodríguez-Diaz, Beatriz Valero, Rosario Sánchez-Martínez, Joaquín Portilla

**Affiliations:** 1 Department of Internal Medicine. General University Hospital of Alicante and Institute for Health and Biomedical Research of Alicante (ISABIAL- Foundation FISABIO), Alicante, Spain; 2 Miguel Hernández University of Elche. San Joan d’Alacant Campus, Alicante, Spain; 3 Infectious Diseases Unit. General University Hospital of Alicante and Institute for Health and Biomedical Research of Alicante (ISABIAL- Foundation FISABIO), Alicante, Spain; 4 Microbiology Service. General University Hospital of Alicante and Institute for Health and Biomedical Research of Alicante (ISABIAL- Foundation FISABIO), Alicante, Spain

**Keywords:** Bacterial infection, Outcomes research

## Abstract

People over 80 years old are now the fastest-growing age group. Bloodstream infections (BSI) in these patients may present with specific characteristics. The objective of this study was to analyze independent factors affecting in-hospital mortality (IHM) due to BSI in very elderly patients (≥80 years of age) and to compare the clinical presentation of BSI in patients aged 80–89 years versus those aged 90 or more. Retrospective, cross-sectional and observational study of BSI in patients aged 80 years or older. The study used IHM as the primary outcome. Stepwise multiple logistic regression models were used to identify associations between potential predictors and IHM. Of the 336 included patients, 76.8% (n = 258) were in the 80–89-year age group and 23.2% (n = 78) in the 90+ age group; 17.3% (n = 58) of patients died during admission. This outcome was independently associated with quick Sepsis Related Organ Failure Assessment (qSOFA) of 2 or more (adjusted odds ratio [aOR] 4.7, 95% confidence interval [CI] 2.3–9.4; p < 0.001). Other predictors included an origin of BSI outside the urinary tract (aOR 5.5, 95% CI 2.4–12.6; p < 0.001), thrombocytopenia (aOR 4.9, 95% CI 1.8–13.4; p = 0.002), hospital-acquired infection (aOR 3.0, 95% CI 1.2–7.5; p = 0.015), and inappropriate empiric antibiotics (aOR 2.0, 95% CI 1.1–3.9; p = 0.04). IHM was 23.1% in the 90+ age group and 15.5% in patients aged 80 to 89 (p = 0.012). However, the 90+ age group was more likely to have a score of at least 2 on the qSOFA (29.9% vs. 19.1%, p = 0.043) and Pitt bacteremia scales (44.9% vs. 30.2%; p = 0.02), as well as chronic kidney disease (56.4% vs. 36.0%; p = 0.001) and altered mental state (40.3% vs. 25.7%; p = 0.013). In conclusion: A qSOFA score of 2 or more and a BSI originating outside the urinary tract were independent predictors of IHM. The 90+ age group was at higher risk than the 80–89-year age group of having a qSOFA score and Pitt bacteremia score of 2 or more as well as an altered mental state.

## Introduction

In recent decades, a lower birthrate and increased life expectancy have contributed to inverting the structure of the population pyramid in Spain and other high-income countries^1,2^. People over 80 are now the fastest-growing age group and are expected to double in number by 2035 in Spain^1^. Improved living conditions, together with advances in health and social care, are the two major drivers of this change.

Bacteremia is a classic marker of severe disease and a common complication of infection in older patients. However, the clinical presentation of older patients with sepsis is often atypical, making diagnosis difficult^3^. Age itself is another confounder linked with a poorer prognosis. Comorbidities, institutionalization, instrumentation, declining functional status, and altered immune function all pose a high risk for bacteremia in older patients^4^. Furthermore, mortality rates for most of these infections are at least three times higher in the elderly compared to younger adults with the same disease^3,5^, and this risk remains higher even after controlling for potential confounders^3,6,7^. Several host factors are described as contributing to increased mortality: age-related state of reduced physiological reserve, underlying chronic diseases, poor response to antimicrobial therapy, and higher rates of adverse reactions to drugs^8^. Other risk factors contributing to higher mortality in elderly patients include a greater risk of hospital-acquired infection^9^, delayed diagnosis and therapy, and initial use of inappropriate antibiotics^4,10^.

Previous studies on this subject include several reviewing the risk factors in BSI and elderly patients^11^; a few comparing BSI in younger, middle-aged, and elderly patients in a general hospital^6,12–14^; and a study that took place in a geriatric hospital. However, there is only limited information concerning the epidemiology, clinical presentations, and prognosis for BSI in patients over 80 years of age^3^, while research in those aged 90 years or older is more limited than in any other age group^2^.

Patients over 90 have higher comorbidities than those aged 80 to 89, and they have been consistently recognized as a group showing poorer outcomes following BSI^15^. Moreover, the clinical presentation of BSI in patients aged 90+ is not well differentiated from all the patients aged over 80^3,7,12,16^.

The quick Sepsis Related Organ Failure Assessment (qSOFA) score is a bedside tool to identify patients with suspected infection outside the intensive care unit who are at greater risk for a poor outcome^17^. The qSOFA score can be used to identify bacteremia patients at high risk of death^17–19^.

The objective of this study was to analyze independent factors affecting in-hospital mortality (IHM) due to BSI in very elderly patients (≥80 years of age), focusing on the domains tested by the qSOFA. A secondary objective was to compare the clinical presentation of BSI in patients aged 80–89 years versus 90+ years.

## Methodology

### Study design and setting

In this retrospective, cross-sectional study, we revised positive blood culture results from the microbiology laboratory in inpatients aged 80 years or older in the General University Hospital of Alicante (Spain) between January 2016 and December 2017 (24 months).

The center is a 750-bed acute care hospital that provides a full range of medical and surgical services to a population that numbered 265,000 in 2017. Total adult admissions were 31,627 patients in 2016 and 30,901 in 2017.

### Selection of participants and data collection

Blood cultures were performed, processed, and interpreted in accordance with the recommendations of the Spanish Society of Infectious Diseases and Clinical Microbiology. Coagulase-negative staphylococci [CoNS], *Bacillus* spp.*, Propionibacterium acnes, Micrococcus luteus, Corynebacterium* spp.*, Lactobacillus* spp.*, Streptococcus viridians* and *Clostridium* spp. were considered contaminant pathogens if they were only in one set of blood cultures. These episodes were excluded^20^.

For each of the 336 episodes of BSI, we collected demographic data (age, sex); length of hospital stay; comorbid conditions (congestive heart failure, chronic kidney disease, malignancy, diabetes mellitus, chronic lung disease, dementia); surgery within 30 days; date of first positive blood culture; and ward of admission at the time of culture (medical, surgery, or other). We also recorded whether or not the patient had a Foley catheter at the time of blood culture, initial vital signs, and routine laboratory test results. On admission and during hospitalization after BSI, we also assessed altered mental state (defined as a Glasgow Coma Scale score under 15 or a decrease of 3 points or more in the presence of a primary central nervous system injury) and acute respiratory distress (defined as pulse oxygen saturation of less than 90% the day of bacteremia)^14^. The hospital information system (Orion Clinic and Mizar) was used to collect analytical and clinical data for inpatients.

The primary outcome of the study was all-cause in-hospital mortality [IHM] in elderly patients with BSI.

### Definitions

Comorbidities were recorded according to the Charlson index, a weighted score that takes into account the number and seriousness of underlying disorders^21^.

We assessed severity of illness the day before the onset of bacteremia using the Pitt bacteremia score, calculated based on temperature (35.1 °C to 36 °C or 39.0 °C to 39.9 °C: 1 point, ≤35 °C or ≥40 °C: 2 points), blood pressure (hypotension: 2 points), mental state (disorientation: 1 point, stupor: 2 points, coma: 4 points), respiratory status (mechanical ventilation: 2 points) and cardiac status (cardiac arrest: 4 points). These are converted to a score on a 4-point scale. We considered a score of 2 or more as conferring a higher risk of mortality^22–24^.

Our definition of severe sepsis at presentation was from the consensus report published by the American College of Chest Physicians and the Society of Critical Care Medicine in 1992. Severe sepsis is indicated by at least two systemic inflammatory response syndrome (SIRS) criteria: core body temperature >38 °C or <36 °C; heart rate ≥90 beats per min; ≥20 breaths per min (or PaCO_2_ <32 mmHg); white blood cell counts of ≥12,000/μL or ≤4000/μL, or >10% in immature forms); plus acute organ dysfunction (including hypoperfusion and hypotension).

The qSOFA uses three criteria, assigning one point each to: low systolic blood pressure (≤100 mmHg), high respiratory rate (≥22 breaths per min), and altered mental state (Glasgow coma scale <15)^19,25^.

Suspected source of infection was determined based on a review of the available progress notes. Categories of infection sources included the lower respiratory tract, urinary tract, biliary tract, intra-abdominal area, catheter-related infections, skin, or ‘other’ source of BSI using Centers for Disease Control and Prevention (CDC) criteria. Those without a localized source of bacteria after an extensive admission work-up were classified as having primary bacteremia^16^.

Each episode of BSI was classified as hospital-acquired infection (>72 h post-admission cutoff for the first positive blood culture or admission in the previous 30 days) or community-acquired using the CDC definition. We sub-classified some community-acquired infections as healthcare-associated infections.

Inappropriate antimicrobial therapy was defined as the lack of antimicrobial therapy for a known pathogen or administration of an antimicrobial agent to which the microorganism was resistant^3^. Polymicrobial bacteremia was defined as either growth of two or more different species of microorganisms in the same blood culture or growth of different species in two or more separate blood cultures for the same case^3,14^. Pathogens were considered multidrug-resistant (MDR) if they showed acquired non-susceptibility to at least one agent in three or more antimicrobial categories^26^.

### Statistical analysis

Data reviewed on each initial BSI per admission were stored on custom-made Excel spreadsheets.

Categorical data were expressed as absolute and relative frequencies. Epidemiological, clinical and microbiological variables in the 80–89-year and 90+ age groups were compared by means of Chi-square tests and Fisher’s exact test (in the case of small numbers). Survivors and deaths were compared by Chi-square and Fisher’s exact test. The two-tailed significance level for all statistical tests was p < 0.05.

We used stepwise multiple logistic regression models to identify associations between potential predictors and IHM. Potential predictors for the regression models were chosen based on clinical importance and results of bivariable analysis; we selected variables showing at least a weak association with in-hospital mortality (p < 0.10), after adjusting for sex and age), using a stepwise selection method with the likelihood ratio test. Model validity was evaluated by the Hosmer-Lemeshow test for estimating goodness of fit to the data and its discriminatory ability using the area under the receiver operating characteristics (ROC) curve (AUC). The magnitude of the effect was quantified as an adjusted odds ratio (aOR) with 95% confidence interval (CI).

The AUC was then used to examine qSOFA discrimination of IHM and compare it to the Pitt bacteremia score and severe sepsis in patients. Sensitivity, specificity, positive predictive value (PPV) and negative predictive value (NPV) were used to determine the best cutoff point in each score. All analyses were carried out on SPSS software version 23.0 (SPSS, Inc., Chicago, IL, USA).

### Ethical aspects

The study was approved by the Ethics Committee of the General University Hospital of Alicante (CEIm PI 2018/105).

### Ethical approval

The Ethical Committee of the General University Hospital of Alicante approved the study (CEIm PI2018/105).

### Informed consent

This is a retrospective study a informed waived has obtained from the Ethical Committee.

## Results

### Rate of BSI in inpatients aged 80 years or older

During the study period, 9333 patients aged 80 years or older were admitted (7183 in the 80–89-year age group and 2150 in the 90+ age group). Blood samples for hemoculture were obtained in 1794 patients (1207 in the 80–89-year age group [16.8%] and 581 [27.0%] in the 90+ age group, p = 0.055). BSI was detected in 336 patients, who we included in our study (258 [21.4%] aged 80 to 89 years, and 78 aged 90+ [pooled proportion: 13.4%], p = 0.1). Table 1 shows the rate of BSI by age and study period. The rate of BSI was 36/1000 admissions in our overall study population, with similar rates in both age groups.

**Table 1:** Episodes of bloodstream infections (BSI) and rate by 1000 admission.

	BSI			N admission			Rate per 1000 admissions		
	2016	2017	Total	2016	2017	Total	2016	2017	Total
**Age group**									
80–89	122 (80.3)	136 (73.9)	258 (76.8)	3592	3591	7183	33.9	37.9	35.9
+90	30 (19.7)	48 (26.1)	78 (23.2)	1004	1146	2150	22.9	41.9	36.3
*Total*	*152*	*184*	*336 (100)*	4596	4737	9333	*33.1*	*38.8*	*36.0*

### Patient characteristics, clinical features, etiology and mortality

Demographic, epidemiological and clinical data are shown in Table 2. With respect to BSI acquisition, 82.7% of episodes were community acquired. The most frequent underlying condition was congestive heart failure (42.6%). Approximately 8% of BSI patients presented urinary catheterization, and 25.3% had taken antibiotics in the previous 3 months. Altered mental state was present in 29.0% of the study population, and low systolic blood pressure (<100 mmHg) in 24.3%.

**Table 2:** Demographic, epidemiological and clinical variables in very elderly patients with bloodstream infections (BSI).

	All patients
Male sex	147 (43.8)
Acquisition	
Community	278 (82.7)
Hospital-acquired infection	36 (10.7)
Healthcare-related	22 (6.5)
Ward of admission	
Medical	284 (84.8)
Intensive care unit	13 (3.9)
Others	39 (11.5)
Severity or comorbidity	
CCI >2	211 (62.8)
Severe sepsis or shock	159 (47.9)
Pitt bacteremia score ≥2	113 (33.9)
qSOFA ≥2	72 (21.6)
Comorbidities	
Congestive heart failure	143 (42.6)
Chronic kidney failure	137 (40.8)
Treated with statins	110 (32.7)
Diabetes mellitus	105 (31.3)
Malignancies	72 (21.4)
Chronic lung disease	76 (22.6)
Dementia	67 (19.9)
Invasive procedures	
Previous surgery	35 (10.4)
Urinary catheterization	28 (8.3)
Nasogastric tube	8 (2.4)
Previous antimicrobial use	85 (25.3)
Clinical manifestations	
Heart rate >90 beats per min	193 (58)
Temperature >38 °C or <36 °C	163 (48.9)
Altered mental state	97 (29.0)
SBP <100 mmHg	81 (24.3)
Respiratory rate >22 breaths per min	63 (19.9)
Acute respiratory distress	57 (17.0)
Analytical	
Lactate >2 mg/L^†^	180 (67.7)
WBC ≥12.0 or ≤4.0 × 103/µL	189 (56.3)
Hemoglobin <11 g/dL	118 (35.1)
Platelet ≤100 × 10^3^/µL	25 (7.4)

^†^Only available in 266 episodes.

Notes: CCI: Charlson comorbidity index; qSOFA: quick Sepsis Related Organ Failure Assessment; SBP: systolic blood pressure; WBC: white blood cells.

The most common origins of BSI were urinary (44.3%, n = 149) and respiratory (20.8%, n = 70) (Table 3). Regarding the etiology of the BSI, 20 of the 336 (6%) episodes were polymicrobial. The most common microorganism was *Escherichia coli* (43.2%, n = 145), followed by *Enterococcus* spp. (12.4%, n = 39) and *Klebsiella* spp. (10.4%, n = 35). Only 4.5% of episodes were caused by CoNS (n = 15) and 3.6% (n = 12) by *Staphylococcus aureus*. Infections were resistant to multiple drugs in 29.5% (n = 99) of the cases. The pathogen was identified in other sources in 44.3% (n = 149) of the episodes, especially in urine (65.8%).

**Table 3:** Source and etiology of bloodstream infections (BSI) in very elderly patients.

	All patients
Source	
Urinary tract	149 (44.3)
Lower respiratory tract	70 (20.8)
Biliary tract	31 (9.29
Primary bacteremia	30 (8.9)
Intra-abdominal	19 (5.7)
Skin and soft tissue	15 (4.5)
Catheter-related infection	16 (4.8)
Other	6 (1.8)
Etiology	
*Escherichia coli*	145 (43.2)
*Enterococcus* spp.	39 (12.4)
*Klebsiella* spp.	35 (10.4)
*Streptococcus viridians*	16 (4.8)
Coagulase-negative staphylococci	15 (4.5)
*Streptococcus pneumoniae*	15 (4.5)
*Proteus mirabilis*	15 (4.5)
Other Enterobacteriaceae^†^	15 (4.2)
*Staphylococcus aureus*	12 (3.6)
*Pseudomonas aeruginosa*	10 (3.0)
Gram-negative bacteria^‡^	9 (2.7)
Other gram-positive bacteria^§^	8 (2.4)
*S. agalactiae*	6 (1.8)
*Candida* spp.	6 (1.8)
Anaerobic bacteria	6 (1.8)
*Streptococcus pyogens*	3 (0.9)
Polymicrobial infections	20 (6.0)
Multidrug resistant bacteria	99 (29.5)
Isolation in other origin	149 (44.3)
Urine	98 (65.8)^‖^
Skin	12 (8.1)^‖^
Catheter	13 (8.7)^‖^
Sputum	7 (4.7)^‖^
Other	19 (12.7)^‖^
Inappropriate empiric antibiotics	104 (31.6)

^†^*Enterobacter spp*. (n = 6); *Providencia stuartii* (n = 2), *Serratia marcenses* (n = 2), *Morganella morganii* (n = 2) *Citrobacter freundii* (n = 2), *Pantoea agglomerans* (n = 1).

^‡^*Campylobacter jejuni* (n = 2), *Haemophylus influenzae* (n = 2), *Pasteurella multocida* (n = 2), *Bordetella holmesii* (n = 1), *Neiseria meningitidis* (n = 1), *Salmonella spp*. (n = 1).

^§^*Actinobaculum urinae* (n = 2), *Micrococus luteus* (n = 2), *Actinobaculum schaalii* (n = 1), *Corynebacterium mucifaciens* (n = 1), *Lactobacillus murinus* (n = 1), *Listeria monocytogenes* (n = 1).

^‖^Percentage of isolation at origin (n = 149).

Empiric antibiotics were inappropriate in 31.6% (n = 104) of episodes. Fifty-eight patients (17.4%) died during admission.

### Clinical features, source and BSI etiology in patients (80–89 years vs. 90+)

Compared to 80–89-year-olds, those aged 90+ showed a significantly higher prevalence of chronic kidney disease (56.4% [n = 44/78] vs. 26.0% [n = 93/258], p = 0.001), Pitt bacteremia score of 2 or more (44.9% [35/78] vs. 30.2% [78/258], p = 0.02), qSOFA of 2 or more (29.9% [23/78] vs. 19.1% [n = 49/258], p = 0.043), respiratory rate of at least 22 breaths per min (28.6% [n = 22/78] vs. 16.0% [n = 41/258], p = 0.013) and altered mental state (31% [n = 31/78] vs. 25.7% [n = 66/258], p = 0.013) on physical examination. There were no differences in other clinical or analytical variables.

When comparing sources and etiology of BSI, the oldest patients more frequently presented lower respiratory tract infection (29.5% [n = 23/78] vs. 18.2% [47/258], p = 0.032) and CoNS (10.3% [n = 8/78] vs. 2.7% [n = 7/258], p = 0.005), while microorganisms in other sources were less commonly isolated (33.3% [26/78] vs. 47.7% [123/258], p = 0.025). Finally, IHM was 23.1% in the 90+ age group and 15.5% in the 80–89-year age group (p = 0.012).

### Analysis of risk factors for mortality in elderly patients

An analysis of the demographic, epidemiological and clinical factors associated with mortality in very elderly patients with BSI is set out in Tables 4, 5. In the bivariable analysis, IHM was significantly higher in patients with a Pitt bacteremia score of 2 or more, severe sepsis, qSOFA of 2 or more, intravenous catheterization, altered mental state (Glasgow coma scale <15), acute respiratory distress, and previous use of antibiotics. Regarding the laboratory analysis, there was higher mortality in patients with anemia and thrombocytopenia. Patients receiving inappropriate antibiotic treatment also had higher IHM.

**Table 4:** Analysis of demographics, risk factors, and clinical factors related to in-hospital mortality in very elderly patients with bloodstream infections (BSI).

		n deaths/N exposed (%)	P value
Sex	Male	26/147 (17.7)	0.9
	Female	32/189 (16.9)	
Age group	80–89 years	40/258 (15.5)	0.12
	90+ years	18 /78 (23.1)	
Hospital-acquired infection	Yes	14/36 (38.9)	<0.001
	No	44/300 (14.7)	
CCI ≥2	Yes	43 /211 (20.4)	0.049
	No	15/125 812.0)	
Pitt bacteremia score ≥2	Yes	32/113 (28.3)	<0.001
	No	26 /223 (11.7)	
Severe sepsis	Yes	41/159 (25.8)	<0.001
	No	15/173 (8.7)	
qSOFA ≥2	Yes	26 /72 (36.1)	<0.001
	No	32/262 (12.2)	
Congestive heart failure	Yes	30/143 (21.0)	0.121
	No	28/193 (14.5)	
Chronic kidney failure	Yes	28/137 (20.4)	0.201
	No	30/199 (15.1)	
Diabetes mellitus	Yes	15/105 (14.3)	0.330
	No	43/231 (18.6)	
Neoplasm	Yes	13/72 (18.1)	0.841
	No	45/ 264 17.0)	
Chronic lung diseases	Yes	42/260 (12.6)	0.320
	No	16/76 (21.1)	
Dementia	Yes	16/67 (23.9)	0.109
	No	42/269 (15.6)	
Treated with statins	Yes	16/110 (14.5)	0.358
	No	42/226 (18.6)	
Urinary catheterization	Yes	5/28 (17.9)	1.0
	No	53/308 (17.2)	
Nasogastric tube	Yes	3/8 (37.5)	0.144
	No	55/328	
Previous surgery	Yes	7/35 (20)	0.651
	No	51/301 (16.9)	
Previous use of antibiotics	Yes	23/85 (27.1)	0.006
	No	35/251 (13.9)	
Temperature >38 °C o < 36 °C	Yes	20/163 (12.2)	0.02
	No	37/170 (21.8)	
Altered mental state	Yes	31 /94 (33.0)	<0.001
	No	27 /241 (11.2)	
Acute respiratory distress	Yes	20/57 (35.1)	<0.001
	No	38/279 (13.6)	
Hemoglobin <11 g/dL	Yes	31/118 (26.3)	0.001
	No	27/216 (12.4)	
White blood cells ≥12.0 × 10^3^/µl	Yes	32/189 (16.9)	0.856
	No	26/147 (17.7)	
Platelet ≤100 × 10^3^/µL	Yes	9/25 (36.0)	0.023
	No	49/311 (15.8)	
Lactate >2 mg/dL	Yes	29/180 (16.1)	0.479
	No	11/86 (12,8)	
Inappropriate empiric antibiotics	Yes	29/104 (27.9)	<0.001
	No	25/225 (11.1)	

Notes: CCI: Charlson comorbidity index, qSOFA: quick sepsis-related organ failure assessment.

**Table 5:** Analysis of etiology and sources related to in-hospital mortality in very elderly patients with bloodstream infections (BSI).

		n deaths/N exposed (%)	P value
*E coli*	Yes	21/175 (12.0)	0.008
	No	37/161 (23.0)	
*Klebsiella* spp.	Yes	7/35 (20.0)	0.6
	No	51/301 (16.9)	
*Pneumococcus*	Yes	4/15 (26.7)	0.3
	No	54/321 (16.8)	
*Enterococcus* spp.	Yes	10/37 (25.6)	0.1
	No	48/297 (16.2)	
Coagulase-negative staphylococci	Yes	8/15 (53.3)	0.001
	No	50/321 (15.6)	
*Candida* spp.	Yes	3/6 (50)	0.066
	No	55 /330 (16.7)	
Anaerobes	Yes	3/6 (50)	0.066
	No	55/330 (16.7)	
Polymicrobial	Yes	7/20 (35.0)	0.06
	No	51 /316 (16.1)	
Multidrug resistant pathogen	Yes	27/99 (27.3)	0.002
	No	31/237 (13.1)	
Urinary tract infection	Yes	9/149 (6.0)	<0.001
	No	49/187 (26.2)	
Lower respiratory tract infection	Yes	20/70 (28.6)	0.005
	No	38 /266 (14.3)	
Biliary tract infection	Yes	3/31 (9.7)	0.3
	No	55/250 (18.0)	
Primary bacteremia	Yes	7/30 (23.3)	0.2
	No	51/306 (16.7)	
Intra-abdominal infection	Yes	7/19 (36.8)	0.029
	No	517317 (16.1)	
Skin infection	Yes	4/15 (26.7)	0.2
	No	54/321 (16.8)	
Catheter-related infection	Yes	4/15 (26.7)	0.3
	No	54/ 321 (16.8)	

IHM rates associated with the source of BSI are shown in Table 5. The lowest IHM rates were for urinary tract (6%) and biliary tract infections (9.7%). The highest mortality rates were seen in intra-abdominal infections outside the biliary tract (36.8%) and lower respiratory tract infections (28.6%).

Regarding the etiology, there was lower IHM for episodes caused by *E. coli* and higher mortality for episodes caused by CoNS. There were only six episodes each caused by a fungus and by an anaerobic microorganism, and three patients in each group died during admission (Table 5). We also analyzed the prevalence of antimicrobial-resistant pathogens and their association with IHM; IHM rates for MDR pathogens were 27.3% and for non-MDR pathogens, 13.1%.

In an exploratory multivariable analysis testing associations with IHM, we introduced the following dichotomous variables into the logistic regression model: age group, sex, Pitt bacteremia score of 2 or more, severe sepsis or shock, qSOFA of 2 or more, hospital-acquired infection, thrombocytopenia, anemia, bacteremia due to *E. coli*, source of BSI other than the urinary tract, and inappropriate antibiotic treatment. We also analyzed urinary tract and lower respiratory tract sources, observing independent associations between IHM and source of BSI other than urinary tract, qSOFA of 2 or more, thrombocytopenia, and inappropriate empiric antibiotics (Table 6). In this model, the p value for the Hosmer-Lemeshow goodness-of-fit test was 0.96, with an AUC of 0.80 (meaning good predictive ability).

**Table 6:** Multivariable analysis of in-hospital mortality in very elderly patients with bloodstream infections (BSI).

	Adjusted OR (95% CI)	P value
Source of BSI other than urinary tract	5.5 (2.4–12.6)	<0.001
qSOFA	4.7 (2.3–9.4)	<0.001
Thrombocytopenia	4.9 (1.8–13.4)	0.002
Hospital-acquired infection	3.0 (1.2–7.5)	0.015
Inappropriate empiric antibiotic	2.0 (1.1–3.9)	0.040

Notes: OR: Odds ratio; CI: confidence interval, qSOFA: quick sepsis-related organ failure assessment.

### Comparison between qSOFA, Pitt bacteremia score and severe sepsis to predict IHM

In these elderly patients, 21.4.% had qSOFA of 2 or more; 33.9%, Pitt bacteremia score of 2 or more; and 47.9%, severe sepsis. The qSOFA, Pitt bacteremia score and severe sepsis did not show good discriminatory ability to predict IHM (AUC <0.37 in all cases) in elderly patients. The performance characteristics of each score are shown in Table 7. The qSOFA outperformed the Pitt bacteremia score in sensitivity, specificity, and PPV, while severe sepsis showed higher sensitivity that the Pitt score, but lower specificity and PPV.

**Table 7:** Performance characteristics of various scores to predict in-hospital mortality in very elderly patients with bloodstream infections (BSI).

Score	n/N exposed (%)	AUC	Sensitivity	Specificity	PPV	NPV
qSOFA ≥2	72/334 (21.4)	0.359	0.448	0.833	0.364	0.877
Pitt bacteremia score 2	113/333 (33.9)	0.366	0.355	0.706	0.283	0.847
Severe sepsis	159/332 (47.9)	0.348	0.732	0.572	0.257	0.913

Notes: AUC: area under receiver operating characteristics curve, PPV: positive predictive value, NPV: negative predictive value, qSOFA: quick sepsis-related organ failure assessment.

## Discussion

This study describes the features of BSI in very elderly patients (80–89 years vs. 90+ years) and identifies predictors of IHM. Some features of the population are noteworthy. In our study, the rate of BSI in the 90+ age group was only slightly higher than in the 80–89-year-old age group, suggesting that very advanced age does not confer a higher risk of BSI in the elderly population. However, there may have been some selection bias rooted in limited diagnostic efforts (including blood cultures) in the very elderly (90+ age group), and a poorer baseline status cannot be ruled out^3^.

In this study, 82.7% of BSI episodes were acquired in the community and 6.5% in a community-healthcare context, which is consistent with some earlier studies^10^.

Regarding co-morbidities, most patients had a Charlson index of more than 2. Previous studies have observed higher Charlson scores in patients with BSI aged 65 to 80 years compared to older patients^3,27^. Most of our patients had comorbidities, including chronic heart failure, chronic kidney failure, diabetes mellitus, malignancies, chronic lung disease and dementia. Epidemiological characteristics of BSI and comorbidities were similar in both age groups studied, with the unsurprising exceptions of chronic kidney disease and dementia. Dementia is also associated with dysphagia, especially in people with moderate to severe cognitive impairment^28^; the dysphagia is related with aspiration and pneumonia, both associated with increased mortality^28^. Chronic kidney disease is also correlated with advancing age. Thus, the higher prevalence seen in our older patients could be a result of their age, although it could also be related to BSI and mortality^29^.

The most frequent source of bacteremia (44.3%) was, predictably, the urinary tract, as in previous studies^4,27,30^. The source of bacteremia was similar in both age groups, but the origin was more frequently in the lower respiratory tract in the 90+ age group. Infections at this site are one of the main causes of admission for infection in very old patients, which could explain our results^31^. Samples other than blood (e.g. urine) were taken less frequently in the oldest group, probably due to limited efforts in these patients^5^.

The urinary tract is the main origin of bacteremia, which is concordant with the fact that *E. coli*, the main microorganism causing bacteremia in elderly patients^27,30,32^, was the most prevalent pathogen, followed by *Enterococcus* spp. and *Kelbsiella* spp.

CoNS were present in 4.5% of samples, and *S. aureus* in 3.6%. In the literature, prevalence in BSI of CoNS ranges from 10% to 30%, and of *S. aureus* from 5% to 10%^33^. In geriatric patients with nosocomial BSIs, CoNS are common, causing around 20% of these infections. Age and hospital admission may influence those results^9^. The microorganisms isolated in the two age groups were similar, with the only difference being the greater presence of CoNS in the older group. As mentioned above, this difference is probably attributable to age^9^.

In our study, 31.6% of patients, with comparable proportions in both groups, received inappropriate empiric antibiotics. These results are in keeping with other studies of BSI in populations aged over 65^3,32^.

The 90+ age group was more likely to have high qSOFA and Pitt bacteremia scores, as well as tachypnea, altered mental state, and low systolic blood pressure. These observations are in concordance with the study by Retamar *et al*., which described altered mental state and tachypnea as the most relevant clinical expressions of bacteremia in patients aged over 90^3^.

In terms of prognosis, IHM was 17.3% overall and slightly higher in the 90+ age group (23.1%) than in the 80–89-year group (15.5%). In the multivariable analysis, acute severity of illness (qSOFA≥ 2) and an origin of bacteremia outside the urinary tract (intra-abdominal or respiratory tract) were strong independent predictors of IHM, as were thrombocytopenia, inappropriate antibiotics, and hospital-acquired infection.

In our patients, the qSOFA score was a better predictor of mortality than severe sepsis or the Pitt bacteremia score in a multivariable logistic regression analysis. This is consistent with a retrospective cohort study in individuals with *Enterobacteriaceae* sepsis receiving appropriate initial antimicrobial therapy^34^. Other studies show that the qSOFA score can be used independently to identify bacteremia patients at higher risk of death, intensive care admission and length of emergency department stay^18,35,36^.

The prognostic value of the qSOFA in very elderly patients is limited^19,37^. While there was an independent association in our sample, in another study (this time in the emergency department setting and involving people aged 75 or older^19^), the qSOFA had little discriminatory power to predict infection-related mortality (with or without bacteremia). However, when the same research group evaluated patients with MDR bacteria, the predictive value improved^37^.

In a cohort of geriatric patients admitted for urinary tract infections, Artero *et al*.^38^ did not observe an association between bacteremia and a worse prognosis; mortality was related to other causes (septic shock and age). Likewise, Honda *et al*.^32^ also failed to find an association between bacteremia and a worse prognosis in elderly patients with pyelonephritis or urinary sepsis. However, BSI in infections of abdominal, respiratory, or unknown origin have been associated with poorer outcomes in several studies^39^, as in ours.

In our study, as elsewhere, mortality was associated with receiving inappropriate antibiotics^3,10,32^. Other studies have reported different risk factors associated with mortality, including chronic kidney disease, hypotension and hypoalbuminemia^10,40^. In our study, we did not find an association with chronic kidney disease, although this was significantly more common in the oldest patients^24^.

In our study, qSOFA, Pitt bacteremia score and severe sepsis did not have good discriminatory power to predict IHM. On the other hand, Battle *et al*.^24^ reported that a quick Pitt score showed good discrimination and performed better than other acute severity of illness scores (qSOFA and SIRS) in predicting mortality following Gram-negative BSI in a patients with a median age of 65 years.

The study has some limitations. First, it used retrospective data collection, which introduces a potential source of information bias and also means that it was not always possible to extract all necessary information about the cases. Secondly, we did not take other explanatory variables into account, such as albumin or immunity status, and we did not consider data on long-term survival and nursing home requirements. Thirdly, our study did not include younger patients with BSI. Due to the dearth of BSI studies in the very elderly, it is difficult to contrast our results with those published in the literature, so there is a continuing need to for larger studies in patients of advanced age. Finally, it is an observational study from a single center, which could limit the generalization of our results.

In conclusion, we observed that high qSOFA and dementia, including altered mental state, were more prevalent in patients aged 90+ than in those aged 80–89 years. Significant predictors of mortality included high qSOFA score, origin of the infection outside the urinary tract, inappropriate empiric antibiotics, thrombocytopenia, and hospital-acquired infection. The use of the qSOFA score for identifying sepsis and administering appropriate empiric antibiotics could contribute to improving outcomes in patients over 80.

### Learning points

- We conducted a clinical comparison of bloodstream infection (BSI) in octogenarians and nonagenarians and identified risk factors for mortality by means of a cross-sectional study.
- Logistic regression analysis showed associations between mortality and qSOFA of 2 or more, a source of infection outside the urinary tract (intra-abdominal or respiratory), presence of thrombocytopenia, inappropriate empiric antibiotics and hospital-acquired infection.
- Very advanced age does not appear to confer a higher risk of BSI in nonagenarians compared to octogenarians.
- The qSOFA can be a useful tool for predicting mortality in octogenarians and nonagenarians with BSI.
